# A Retrospective Chart Review of Pediatric Complicated Community-Acquired Pneumonia: An Experience in the Al Qassimi Women and Children Hospital

**DOI:** 10.7759/cureus.31119

**Published:** 2022-11-05

**Authors:** Sinan Yavuz, Amal Sherif, Maryam Amirrad, Khalid Sabet, Mohamed Hassan, Motasem Abuelreish, Noura Langawi, Mina Almanasir, Nader Francis

**Affiliations:** 1 Pediatrics, Al Qassimi Women’s and Children’s Hospital, Sharjah, ARE; 2 Pediatric Surgery, Al Qassimi Women’s and Children’s Hospital, Sharjah, ARE; 3 Pediatric Infectious Diseases, Al Qassimi Women’s and Children’s Hospital, Sharjah, ARE; 4 Pediatric Pulmonology, Al Qassimi Women’s and Children’s Hospital, Sharjah, ARE

**Keywords:** complicated community-acquired pneumonia (ccap), community-acquired pneumonia (cap), pediatric pneumonology, video-assisted thoracoscopic surgery (vats), necrotizing pneumonia, pleural empyema, parapneumonic effusion

## Abstract

Background

Community-acquired pneumonia (CAP) is one of the most common global health issues. Even though many vaccinations and new diagnostic tools are available, CAP has a higher mortality rate, especially in children less than five years of age. Complicated CAP (CCAP) in a healthy child is a severe disease characterized by a combination of local complications, such as parapneumonic effusion (PPE), empyema (EMP), necrotizing pneumonia (NP), abscess, pneumothorax, and bronchopleural fistula, and systemic complications, such as bacteremia, metastatic infection, multiorgan failure, acute respiratory distress syndrome, disseminated intravascular coagulation, and, rarely, death. This study describes the demographic features, clinical presentation, management, and outcomes of patients diagnosed with CCAP at the Al Qassimi Women’s and Children’s Hospital (AQWCH).

Methodology

This retrospective chart review aims to collect and explore the data of all previously healthy children admitted with CCAP between the ages of one month and 13 years at AQWCH from January 2018 to December 2020. The primary study outcome measure is to provide clinicians with the diagnostics, evaluation, and management required to treat complicated pneumonia.

Results

A total of 195 patients were diagnosed with CAP, of whom 30 (15.3%) were diagnosed with CCAP. Of these, 14 (46.6%) patients had NP, eight (26.7%) had PPE, and eight (26.7%) had EMP. The median age of patients was 2.5 years, with 13 (43%) males and 17 (57%) females. The median duration of their stay in the hospital was 16 days. All patients were vaccinated with Hib, PCV13, or PCV7, and 57% of the patients received antibiotics before admission. The most common findings were consolidation and pleural effusion. Blood culture was negative in all cases, and pleural culture was positive only in three cases. A total of 17 (57%) patients underwent video-assisted thoracoscopic surgery (VATS), and post-VATS surgical emphysema was found to be the most common complication. Chest X-rays normalized after three months in 65% of patients.

On comparing patients who were admitted to the Pediatric Intensive Care Unit (PICU) before any surgical intervention with those who were not, it was found that patients who required PICU admission were young (median = 2 years; interquartile range (IQR) = 1-4.5; p = 0.044) and had higher respiratory rate (mean = 49 breaths/per minute, standard deviation (SD) = 11; p = 0.000). In addition, they had lower median albumin (median = 2 g/L; IQR = 1.8-2.23; p = 0.004).

On comparing patients who required VATS and those who did not require VATS, it was found that the former had a higher median respiratory rate (48 per min; range = 42-54; p = 0.01). A cavity in the chest computed tomography (CT) was found in 86% of patients with VATS (p = 0.017), and they had lower median albumin (median = 2 g/L; IQR = 1.92-2.24; p = 0.012), as well as longer median duration of using oral antibiotics (median = 21 days; IQR = 19-26; p = 0.025).

Patients with complicated NP had a higher respiratory rate and higher PICU admission, and more cavity in the chest was found in the CT study. Most NP patients also underwent VATS and had longer median days of using oral antibiotics. One patient developed a bronchopleural fistula, and one patient diagnosed with NP died.

Conclusions

CCAP is a major cause of hospitalization in children. It is important to suspect CCAP in all CAP patients not responding to treatment after 48-72 hours.

## Introduction

Pneumonia in previously healthy children caused by infection outside the hospital is defined as community-acquired pneumonia (CAP) [1]. CAP remains the largest single cause of morbidity and mortality worldwide in children aged between 28 days (i.e., outside the neonatal period) and five years [2]. Complicated CAP (CCAP) in a previously healthy child is a severe disease characterized by a combination of local complications, such as parapneumonic effusion (PPE), empyema (EMP), necrotizing pneumonia (NP), abscess, pneumothorax, and bronchopleural fistula, and systemic complications, such as bacteremia, metastatic infection, multiorgan failure, acute respiratory distress syndrome, disseminated intravascular coagulation, and, rarely, death [3]. Pleural effusion undergoes three stages, namely, the exudative stage (fluid is serous and sterile), the fibropurulent stage after one to two weeks (inflow of white blood cells and bacteria), EMP over two to four weeks, and progressive thick fibrinous peel (pus formation) [4]. Even though the exact pathology of NP is unknown, it is characterized by severe lung tissue destruction and disintegration [3]. Many studies have shown that genetic predisposition, vascular thrombosis, and vascular occlusion are possible mechanisms of NP [5-7]. The process begins with consolidation and necrosis and then progresses to cavitation, which may convert into a large cyst. If ruptured, it can cause bronchopulmonary fistula [7,8].

The symptoms of CCAP are fever, cough, tachypnea, distress, chest pain, and abdomen and/or shoulder pain. Decreased air entry, dullness, and fine crackles are some of the findings on chest examination. *Streptococcus pneumonia*, *Staphylococcus aureus*, including methicillin-resistant *S. aureus*, and *Streptococcus pyogenes* are the causative organisms [9]. Less common causes include *Haemophilus influenzae*, *Mycoplasma pneumoniae*, and *Pseudomonas aeruginosa*. Children with CCAP have an extended hospitalization duration, radiological evaluation, antibiotic treatment, and analgesic or sedative medications and require more invasive interventions such as chest tube insertion, open decortication, and video-assisted thoracoscopic surgery (VATS), with most patients recovering completely. Systemic complications of CCAP include sepsis and septic shock, metastatic infection, multiorgan failure, acute respiratory distress syndrome, disseminated intravascular coagulation, and death [3].

This retrospective study aims to review the cases of CCAP among children hospitalized at the Al Qassimi Women’s and Children’s Hospital (AQWCH) over a selected time period and to describe the demographic features, clinical presentation, epidemiology, etiology, management, and outcome of patients.

## Materials and methods

Study design

This study is a retrospective chart review. A timeframe sample and de-identified medical record data of all CCAP patients admitted between January 01, 2018, and December 31, 2020, were used. This study was conducted at AQWCH, UAE. All selected patients are minors; however, all data were de-identified, anonymous, and stored on password-protected computers accessed by the primary investigator (PI) only. Ethical approval was obtained from MOHAP REC (approval number: MOHAP/DXB-REC/OOO/No.139/2020).

Study enrollment

The study population needed to be defined using inclusion and exclusion criteria. Of the 195 patients diagnosed with pneumonia, 30 fulfilled the inclusion criteria. Previously healthy children between one month and 13 years of age, admitted in the study timeframe, with a final diagnosis of CCAP were included in the study. Children with chronic lung diseases, neurodegenerative diseases, muscular dystrophies, malnutrition, lung obstruction due to unnoticed foreign bodies, and congenital or acquired immunodeficiencies were excluded.

Statistical methods

The primary endpoint was a descriptive dataset of patients with CCAP who were admitted to AQWCH in the identified time frame. The secondary endpoints explored the characteristics and factors associated with CCAP in children, recognizing their clinical presentation, gaining better knowledge of their management, and improving outcomes of children diagnosed with CCAP at AQWCH.

Statistical analysis

Descriptive statistics were used to describe the characteristics of the variables using frequencies for categorical variables. Data for categorical variables were tested using the chi-square test and Fisher’s exact test. Continuous variables were tested using the t-test and Mann-Whitney U test. The normality was tested using the Shapiro-Wilk test and visualization of histograms. As continuous variables, such as age, were not normal, the Kruskal-Wallis test was performed. The results are shown as tables or diagrams (bar and line graph). The alpha value p ≤ 0.05 was used to determine statistical significance.

## Results

**Figure 1:**
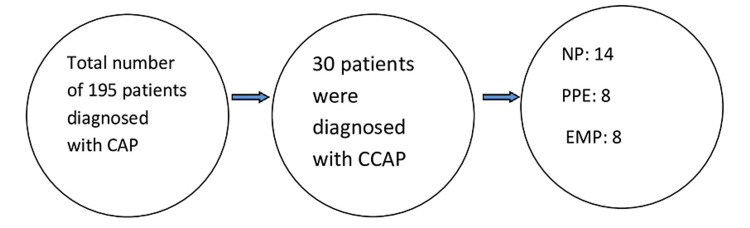
Patient population. CAP: community-acquired pneumonia; CCAP: complicated community-acquired pneumonia; NP: necrotizing pneumonia; PPE: parapneumonic effusion; EMP: empyema

Of the 195 patients diagnosed with CAP, only 30 (15.3%) were diagnosed with CCAP. Of these, NP was diagnosed in 14 (46.6%), PPE in eight (26.7%), and EMP in eight (26.7%) patients (Figure 1).

Demographic and clinical characteristics

This retrospective chart review study consisted of 30 patients with a median age of 2.5 years (range = 1-6 years); 11 (37%) were Emirati and 19 (63%) were non-Emirati, of whom 13 (43%) were males and 17 (57%) were females. Patients were presented with a mean respiratory rate of 44 breaths per minute (13%), mean heart rate of 145 mmHg (24%), mean oxygen saturation of 95% (3%), fever of 29 (97%) with a mean 38.6°C (0.6%), and the median duration of fever before admission was six days (range = 4-7). Cough was commonly present in all patients (100%), chest pain in five (17%), abdominal pain in four (13%), chest recession in 22 (73%), chest dullness in 12 (40%), reduced air entry in the lungs in 29 (97%), and lung crackles in 26 (87%). No patients had a personal or family history of lung disease, immunodeficiency, cystic fibrosis, or failure to thrive; all patients (100%) received Hib, PCV7, or PCV13 vaccines; and two (7%) patients had pneumonia previously. In total, 17 (57%) patients had used antibiotics prior to admission, and eight (27%) were referred from other health facilities; 21 (70%) patients were admitted to the Pediatric Intensive Care Unit (PICU), of whom 14 (67%) were admitted before surgery and seven (33%) were admitted post-surgery (Table 1).

**Table 1:** The demographic, common presenting signs and symptoms, and characteristics in children with CCAP. IQR: interquartile range; CCAP: complicated community-acquired pneumonia; PICU: Pediatric Intensive Care Unit; Hib vaccine: *Haemophilus influenzae* type B vaccine; PCV7: seven-valent pneumococcal conjugate vaccine; PCV13: 13-valent pneumococcal conjugate vaccine

Frequency	Overall study
Demographic characteristics of patients	
Nationality − number (%)	
Emirati	11 (37)
Non-Emirati	19 (63)
Gender − number (%)	
Male	13 (43)
Female	17 (57)
Age (years)	
Median	2.5
IQR	1–6
Characteristics of patients with CCAP	
Previous Hib vaccination − number (%)	30 (100)
Previous PCV7 or PCV13 vaccination − number (%)	30 (100)
Previous pneumonia − number (%)	2 (7)
Use of antibiotic before admission − number (%)	17 (57)
Referral health facility − number (%)	8 (27)
PICU admission − number (%)	21 (70)
Percentage of patients with CCAP who required PICU admission − number (%)	
Pre-operation	14 (67)
Post-operation	7 (33)
Presenting symptoms and signs	
Fever − number (%)	29 (97)
Cough − number (%)	30 (100)
Chest pain − number (%)	5 (17)
Abdominal pain − number (%)	4 (13)
Chest recession − number (%)	22 (73)
Chest dullness − number (%)	12 (40)
Reduced air entry in the lungs − number (%)	29 (97)
Lung crackles − number (%)	26 (87)
Respiratory rate (per minute)	
Mean	44
SD	13
Heart rate (per minute)	
Mean	145
SD	24
Oxygen saturation (%)	
Mean	95
SD	3
Degree of fever (℃)	
Mean	38.6
SD	0.6
Duration of fever (days)	
Median	6
IQR	4–7
Duration of stay in the hospital (days)	
Median	16
IQR	9–19

Radiologic, hematologic, and microbiologic findings on admission

Radiological findings in patients at the time of admission were recorded. The chest X-rays of 30 (100%) patients were obtained, and the findings were consolidation in 30 (100%), pleural effusion in 25 (83%), a cavity in two (7%), and pneumothorax in one (3%). The chest ultrasound was obtained for 27 (90%) patients, and the findings were consolidation in 27 patients (100%), pleural effusion in 24 (89%), a cavity in seven (26%), and septation in 10 (37%). The chest computed tomography (CT) study was performed for 25 (83%) patients, and the findings were consolidation in 25 (100%), pleural effusion in 22 (88%), a cavity in 16 (64%), pneumothorax in two (8%), septation in 12 (48%), and necrotic findings in eight (38%).

The blood test results on admission showed that the median white blood cell count was 17.7 per/mL (interquartile range (IQR) = 14-24), levels of C-reactive protein increased in all patients with a median of 233 mg/L (IQR = 90-300), and the median albumin content was 2.2 g/L (IQR = 1.96-2.74). The blood culture was negative in all cases, and the pleural culture was positive only in three (14%) cases. The isolated organisms were *Staphylococcus haemolyticus*, *S. pneumoniae*, and *S. pyogenes* (Table 2).

**Table 2:** The radiologic, hematologic, and microbiologic findings in patients with CCAP. IQR: interquartile range; CT: computed tomography; CCAP: complicated community-acquired pneumonia

Variables	Frequency
Radiologic findings on admission	
Chest X-ray study − number (%)	30 (100)
Consolidation on the chest X-ray study − number (%)	30 (100)
Pleural effusion on the chest X-ray study − number (%)	25 (83)
Cavity on the chest X-ray study − number (%)	2 (7)
Pneumothorax on the chest X-ray study − number (%)	1 (3)
Chest ultrasound − number (%)	27 (90)
Consolidation on the chest ultrasound study − number (%)	27 (100)
Pleural effusion on the chest ultrasound study − number (%)	24 (89)
Cavity on the chest ultrasound study − number (%)	7 (26)
Septation on the chest ultrasound study − number (%)	10 (37)
Chest CT study − number (%)	25 (83)
Consolidation on the chest CT study − number (%)	25 (100)
Pleural effusion on the chest CT study − number (%)	22 (88)
Cavity on the chest CT study − number (%)	16 (64)
Pneumothorax on the chest CT study − number (%)	2 (8)
Septation on the chest CT study − number (%)	12 (48)
Necrotic findings on the chest CT study − number (%)	8 (38)
Hematologic and microbiologic findings	
White blood cell (per μL)	
Median	17.7
IQR	14–24
C-reactive protein (mg/L)	
Median	233
IQR	90–300
Albumin (g/L)	
Mean	1.3
SD	0.5
Positive pleural culture − number (%)	3 (14)

Follow-up investigations and medical and surgical management

The duration of hospital stay was 16 days (IQR = 8-20), and the duration of hospitalization post-VATS was eight days (IQR = 7-15.5); 22 (73%) patients were required to change their antibiotics during hospitalization; 27 (90%) were discharged on oral antibiotics, the mean duration of oral antibiotics was 18 days (SD = 8), and the median duration of intravenous (IV) antibiotics was 14 days (IQR = 11-21). In total, 17 (57%) patients underwent VATS, and the median time of VATS was six days (IQR = 2-13). The median duration for the removal of the chest tube post-VATS was five days (IQR = 4-11), and the median time for the removal of chest tube insertion without VATS was two days (IQR = 1-3). Two (50%) patients who had only the chest tube required VATS later. Chest taping was performed for three (10%) patients. The complications that occurred in patients who underwent VATS were surgical emphysema in 11 (65%) patients, pneumothorax in four (24%), and bronchopulmonary fistula in one (6%).

Follow-up chest X-rays after three months of treatment were obtained for 17 (57%) patients, and the findings were normal in 11 (65%) patients; however, consolidation was found in five (29%) patients, and one (6%) patient had a cavity. Chest X-rays after six months were obtained for five (17%) patients, and the finding was normal in all patients (100%) (Table 3).

**Table 3:** The follow-up investigations, medical, and surgical management of children with CCAP. IQR: interquartile range; CRP: C-reactive protein; IV antibiotics: intravenous antibiotics; VATS: video-assisted thoracoscopic surgery; CCAP: complicated community-acquired pneumonia

Frequency	Statistics
Duration of stay in the hospital (days)	
Median	16
IQR	8–20
The initial antibiotics changed during hospitalization − number (%)	22 (73)
Oral antibiotics given on discharge − number (%)	27 (90)
Duration of IV antibiotics (days)	
Median	14
IQR	11–21
Duration of oral antibiotic (days)	
Mean	18
SD	8
Patient with VATS − number (%)	17 (57)
Post-VATS surgical emphysema − number (%)	11 (65)
Post-VATS pneumothorax – no (%)	4 (24)
Post-VATS bronchopulmonary fistula − number (%)	1 (6)
Time of VATS (days)	
Median	6
IQR	2–13
Time of chest tube removal (without VATS) (days)	
Median	2
IQR	1–3
Duration of chest tube removal (days)	
Median	5
IQR	4–11
Chest taping − number (%)	4 (13)
Follow-up chest X-ray after 3 months − number (%)	17 (57)
Normal findings on the follow up chest number -ray after 3 months − number (%)	11 (65)
Consolidation on the follow up chest X-ray after 3 months − number (%)	5 (29)
Cavity on the follow up chest X-ray after 3 months − number (%)	1 (6)
Follow-up chest X-ray after 6 months − number (%)	5 (17)
Normal findings on the follow up chest X-ray after 6 months − number (%)	5 (100)

On comparing the different types of CCAP, demographic and clinical presentations were non-significant, except that the respiratory rate was higher in patients with NP (48 breaths/minute vs. 43 EMP and 35 PPE; p = 0.038). Regarding PICU admission, it was found that NP had the highest percentage (93% vs. 75% EMP and 25% PPE; p = 0.004). In the radiological findings, some significant frequencies were similar such as septation on chest ultrasound, with both NP and EMP (50% vs. 0% PPE; p = 0.029), and most patients with NP had a cavity (92% vs. 43% in EMP and 0% PPE; p = 0.000) and necrotic finding on the chest CT study (62% vs. 0% EMP and PPE; p = 0.004). Interestingly, septation on the chest CT study was higher in EMP (74% vs. 54% NP and 0% PPE; p = 0.0047). The median duration of IV antibiotics was higher in EMP (23 days vs. 19 days NP and 10 days PPE; p = 0.02). However, the median duration of oral antibiotics was found to be longer in NP (21 days vs. 18 days EMP and 10 days PPE; p = 0.002). In total, 96% of NP patients had VATS, followed by 38% EMP, and the PPE was 13% (p = 0.002). There were no statistically significant differences in the remaining variables between the three groups (Table 4).

**Table 4:** The findings in the different subgroups of CCAP. NP: necrotizing pneumonia; PPE: parapneumonic effusion; EMP: empyema; IQR: interquartile range; CRP: C-reactive protein; WBC: white blood cells; CT: computed tomography; VATS: video-assisted thoracoscopic surgery; PICU: Pediatric Intensive Care Unit; IV antibiotics: intravenous antibiotics; CCAP: complicated community-acquired pneumonia

Variables	NP	PPE	EMP	P-value
Demographic and clinical characteristics				
Age (years)				0.066
Median	2	6.5	2	
IQR	1–5	3.5–9.5	1–3	
Gender − number (%)				0.414
Male	8 (57)	2 (25)	3 (38)	
Female	6 (43)	6 (75)	5 (62)	
Fever − number (%)				0.54
Yes	14(100)	8 (100)	7 (88)	
No	0 (0)	0 (0)	0 (12)	
Chest pain − number (%)				0.837
Yes	2 (14)	2 (25)	1 (13)	
No	12 (86)	6 (75)	7 (87)	
Abdominal pain − number (%)				0.837
Yes	1 (7)	2 (25)	1 (13)	
No	13 (93)	6 (75)	7 (87)	
Chest recession − number (%)				0.61
Yes	13 (93)	5 (63)	4 (50)	
No	1 (7)	3 (37)	4 (50)	
Chest dullness − number (%)				0.891
Yes	5 (36)	4 (50)	3 (38)	
No	9 (64)	4 (50)	5 (62)	
Reduced air entry in the lungs − number (%)				0.533
Yes	14(100)	8 (100)	7 (88)	
No	0 (0)	0 (0)	1 (12)	
Lung crackles − number (%)				0.787
Yes	13 (93)	7 (88)	6 (75)	
No	1 (7)	1 (12)	2 (25)	
Respiratory rate (per minute)				0.038
Median	48	35	43	
IQR	40–57	28–41	33–54	
Oxygen saturation (%)				0.249
Median	96	98	96	
IQR	94–97	96–99	93–98	
Duration of fever, days				0.197
Median	6.5	6	4	
IQR	5–10	4–7	3–7	
Degree of fever (℃)				0.515
Median	38.6	39.1	38.5	
IQR	38.1–39	38.3–39.2	38–39.2	
Use of antibiotic before admission − number (%)				0.414
Yes	6 (43)	6 (75)	5 (63)	
No	8 (57)	2 (25)	3 (37)	
Referral health facility − number (%)				0.671
Yes	5 (36)	1 (13)	2 (25)	
No	9 (64)	7 (87)	6 (75)	
PICU admission − number (%)				0.004
Yes	13 (93)	2 (25)	6 (75)	
No	1 (7)	6 (75)	2 (25)	
Time of admission to PICU − number (%)				0.89
Pre-operation	8 (57)	0 (0)	5 (63)	
Post-operation	6 (43)	8 (100)	3 (37)	
Radiologic findings on admission				
Pleural effusion on the chest X-ray study − number (%)				0.837
Yes	12 (86)	6 (75)	7 (88)	
No	2 (14)	2 (25)	1 (12)	
Pneumothorax on the chest X-ray study − number (%)				1.00
Yes	1 (7)	0 (0)	0 (0)	
No	13 (93)	8 (100)	8 (100)	
Pleural effusion on the chest ultrasound study − number (%)				0.770
Yes	10 (83)	7 (88)	7 (100)	
No	2 (17)	1(12)	0 (0)	
Cavity on the chest ultrasound study − number (%)				0.325
Yes	5 (42)	1 (14)	1 (14)	
No	9 (58)	7 (86)	7 (86)	
Septation on the chest ultrasound study − number (%)				0.029
Yes	6 (50)	0 (0)	4 (50)	
No	6 (50)	8 (100)	4 (50)	
Pleural effusion on the chest CT study − number (%)				1.000
Yes	11 (85)	5 (100)	6 (86)	
No	2 (15)	0 (0)	1 (14)	
Cavity on the chest CT study − number (%)				0.000
Yes	12 (92)	0 (0)	3 (43)	
No	1 (8)	5 (100)	4 (57)	
Pneumothorax on the chest CT study − number (%)				1.000
Yes	1 (8)	0 (0)	1 (14)	
No	12 (92)	5 (100)	6 (86)	
Septation on the chest CT study − number (%)				0.047
Yes	7 (54)	0 (0)	5 (74)	
No	6 (46)	5 (100)	2 (26)	
Necrotic findings on the chest CT study − number (%)				0.004
Yes	8 (62)	0 (0)	0 (0)	
No	5 (38)	5 (100)	7 (100)	
WBC (per μL)				0.82
Median	21	15	17	
IQR	16–29	7.4–19	9.9–21.8	
CRP (mg/L)				0.209
Median	253	127	186	
IQR	98 -327	34–258	78–306	
Albumin (g/L)				0.169
Median	2	2.7	2.2	
IQR	1.9–2.3	2.2–3.2	1.5–3.2	
Medical and surgical management				
The initial antibiotic changed during hospitalization − number (%)				1.000
Yes	2 (15)	0 (0)	1 (25)	
No	11 (85)	4 (100)	3 (75)	
Oral antibiotic given on discharge − number (%)				0.421
Yes	13 (93)	6 (75)	8 (100)	
No	1 (7)	2 (25)	0 (0)	
Duration of IV antibiotics (days)				0.02
Median	19	10	23	
IQR	14–21	6–14	11–27	
Duration of oral antibiotics (days)				0.03
Median	21	10	18	
IQR	16–20	10–14	13–26	
Patient have VATS − number (%)				0.002
Yes	12 (86)	0 (0)	4 (50)	
No	2 (14)	8 (100)	4 (50)	
Time of VATS (days)				0.825
Median	6	0	10	
IQR	2–13	0	6–13	
Duration of hospitalization				0.164
Median	13	10	21	
IQR	9–19	5–17	10–30	
Duration of hospitalization post-VATS				0.423
Mean	10	0	12	
SD	5	0	6	
Post-VATS surgical emphysema − number (%)				1.000
Yes	8 (67)	0 (0)	2 (67)	
No	4 (33)	0 (100)	1 (33)	
Post-VATS pneumothorax − number (%)				0.365
Yes	3 (25)	0 (0)	0 (0)	
No	9 (75)	0 (0)	3 (100)	
Post-VATS bronchopulmonary fistula − number (%)				1.000
Yes	1 (8)	0 (0)	0 (0)	
No	11 (92)	0 (100)	3 (100)	
Normal findings on the follow up chest X-ray after 3 months − number (%)				1.000
Yes	7 (64)	3 (75)	1 (50)	
No	4 (36)	1 (25)	1 (50)	
Lung consolidation on the follow up chest X-ray after 3 months − number (%)				1.000
Yes	3 (27)	1 (25)	1 (25)	
No	8 (73)	3 (75)	1 (50)	

On testing for association between patients who required PICU admissions and who did not require it, along with other variables, a statistical significance was found (p < 0.05) with age, respiratory rate, and albumin content. Patients who were admitted to PICU had a lower median age of two years (IQR 1-4.5, p = 0.044), a higher mean respiratory rate of 49 beats/minute (SD = 11; p = 0.000), and a lower median albumin content of 2 mg/L (IQR = 1.8-2.23; p = 0.004). There were no significant differences in other frequencies (Table 5).

**Table 5:** The characteristics of patients with CCAP who required PICU admission. IQR: interquartile range; CRP: C-reactive protein; CT: computed tomography; PICU: Pediatric Intensive Care Unit; WBC: white blood cells; IV antibiotics: intravenous antibiotics; CCAP: complicated community-acquired pneumonia

Frequency	PICU admission		P-value
	Yes	No	
Demographic and clinical characteristics			
Age (years)			0.044
Median	2	6	
IQR	1–4.5	2.5–8	
Gender − number (%)			0.229
Male	11 (52)	2 (22)	
Female	10 (48)	7 (78)	
Fever − number (%)			1.00
Yes	20 (95)	9 (100)	
No	1 (100)	0 (0)	
Chest pain − number (%)			0.622
Yes	3 (14)	2 (22)	
No	18 (86)	7 (78)	
Abdominal pain − number (%)			0.563
Yes	2 (10)	2 (22)	
No	19 (90)	7 (78)	
Chest recession − number (%)			0.195
Yes	17 (81)	5 (57)	
No	4 (19)	4 (43)	
Chest dullness − number (%)			0.418
Yes	7 (33)	5 (57)	
No	14 (67)	4 (44)	
Reduced air entry in the lungs − number (%)			1.00
Yes	20 (95)	9 (100)	
No	1 (5)	0 (0)	
Lung crackles − number (%)			0.287
Yes	17 (81)	9 (100)	
No	4 (19)	0 (0)	
Respiratory rate (per minute)			0.000
Median	49	31	
IQR	11	7	
Oxygen saturation (%)			0.144
Median	96	97	
IQR	93–98	96.5–98.5	
Use of antibiotic before admission − number (%)			0.691
Yes	11 (52)	6 (67)	
No	10 (48)	3 (33)	
Radiologic Findings on Admission			
Pleural effusion on the chest X-ray study − number (%)			0.622
Yes	18 (86)	7 (78)	
No	3 (14)	2 (32)	
Pneumothorax on the chest X-ray study − number (%)			1.00
Yes	1 (5)	0 (0)	
No	20 (85)	9 (100)	
Pleural effusion on the chest ultrasound study − number (%)			1.00
Yes	16 (89)	8 (89)	
No	2 (11)	1 (11)	
Cavity on the chest ultrasound study − number (%)			0.363
Yes	6 (33)	1 (11)	
No	12 (67)	8 (89)	
Septation on the chest ultrasound study − number (%)			1.00
Yes	7 (39)	3 (33)	
No	11 (61)	6 (67)	
Pleural effusion on the chest CT study − number (%)			0.534
Yes	15 (83)	7 (100)	
No	3 (17)	0 (0)	
Cavity on the chest CT study − number (%)			0.058
Yes	14 (78)v	2 (29)	
No	4 (22)	5 (71)	
Pneumothorax on the chest CT study − number (%)			1.00
Yes	2 (11)	0 (0)	
No	16 (89)	7 (100)	
Septation on the chest CT study − number (%)			1.00
Yes	9 (50)	3 (43)	
No	9 (50)	4 (57)	
Necrotic findings on the chest CT study − number (%)			1.00
Yes	7 (39)	1 (14)	
No	11 (61)	6 (86)	
Hematologic and microbiologic findings			
WBC (per μL)			0.067
Median	17.9	12.6	
IQR	16–25.5	7.5–23.2	
CRP (mg/L)			0.129
Median	258	77	
IQR	140–304	43–300	
Albumin (g/L)			0.004
Median	2	2.8	
IQR	1.8–2.23	2.4–3.26	
Medical and surgical management			
The initial antibiotic changed during hospitalization − number (%)			0.666
Yes	16 (76)	6 (67)	
No	5 (24)	3 (33)	
Oral antibiotic given on discharge − number (%)			1.00
Yes	19 (90)	8 (89)	
No	2 (10)	1 (11)	
Duration of IV antibiotics (days)			0.011
Median	20	10	
IQR	13–25	6–17	
Duration of oral antibiotics (days)			0.023
Median	21	12	
IQR	14–27	10–14	
Duration of hospitalization (days)			0.65
Median	20	12	
IQR	14–26	6–22	

On testing variables for the association between patients who required VATS and those who did not require VATS, a significant (p < 0.05) association between patients who required VATS and their clinical, laboratory, radiological findings, and management was found. Clinically, there was a significantly higher respiratory rate of 48 breaths/minute (IQR = 42-55; p = 0.01] in patients with VATS. In total, 12 (86%) patients (p = 0.017) who underwent VATS had a cavity in their chest CT scan on admission with no differences in other frequencies. All laboratory tests were equally abnormal in all patients in both groups except for albumin content, which was remarkably lower in patients with VATS (median = 2 mg/L; IQR = 1.2-2.2; p = 0.012). Patients with VATS were found taking oral antibiotics for a longer duration (median = 21 days; IQR = 19-26; p = 0.025], with no remarkable changes in other findings (Table 6).

**Table 6:** The association between patients who required VATS and those who did not require VATS. IQR: interquartile range; CRP: C-reactive protein; CT: computed tomography; VATS: video-assisted thoracoscopic surgery; PICU: Pediatric Intensive Care Unit; WBC: white blood cells; IV antibiotics: intravenous antibiotics

Variables	VATS		P-value
	Done	Not done	
Demographic and clinical characteristics			
Age (years)			0.170
Median	2	4	
IQR	0.170	1–8	
Gender − number (%)			0.159
Male	9 (56)	4 (29)	
Female	7 (44)	10 (71)	
Fever − number (%)			0.467
Yes	16 (100)	13 (93)	
No	0 (0)	1 (7)	
Chest pain − number (%)			1.00
Yes	3 (19)	2 (14)	
No	13 (81)	12 (86)	
Abdominal pain − number (%)			1.00
Yes	2 (13)	2 (14)	
No	14 (87)	12 (86)	
Chest recession − number (%)			0.417
Yes	13 (81)	9 (64)	
No	3 (19)	5 (36)	
Chest dullness − number (%)			0.073
Yes	4 (25)	8 (57)	
No	12 (75)	6 (43)	
Reduced air entry in the lungs − number (%)			1.00
Yes	15 (94)	14 (100)	
No	1 (6)	0 (0)	
Lung crackles − number (%)			0.315
Yes	15 (94)	11 (79)	
No	1 (6)	3 (21)	
Respiratory rate (per minute)			0.01
Median	48	35	
IQR	42–55	28–45	
Degree of fever (℃)			0.351
Mean	38.7	38.5	
SD	0.62	0.56	
Use of antibiotic before admission − number (%)			0.491
Yes	10 (63)	7 (50)	
No	6 (41)	7 (50)	
Time of PICU admission, number (%)			0.123
Pre-operation	9 (56)	5 (100)	
Post-operation	7 (44)	0 (0)	
Radiologic findings on admission			
Pleural effusion on the chest X-ray study − number (%)			0.642
Yes	14 (88)	11 (79)	
No	2 (12)	3 (21)	
Cavity on the chest X-ray study, number (%)			0.209
Yes	0 (0)	2 (14)	
No	16 (100)	12 (86)	
Pneumothorax on the chest X-ray study − number (%)			0.467
Yes	0 (0)	1 (7)	
No	16 (100)	13 (93)	
Pleural effusion on the chest ultrasound study − number (%)			0.596
Yes	13 (93)	11 (85)	
No	1 (7)	2 (5)	
Cavity on the chest ultrasound study − number (%)			0.385
Yes	5 (36)	2 (15)	
No	9 (64)	11 (85)	
Septation on the chest ultrasound study − number (%)			0.695
Yes	6 (43)	4 (31)	
No	8 (57)	9 (69)	
Pleural effusion on the chest CT study − number (%)			0.565
Yes	13 (93)	9 (82)	
No	1 (7)	2 (18)	
Cavity on the chest CT study − number (%)			0.017
Yes	12 (86)	4 (36)	
No	2 (14)	7 (64)	
Pneumothorax on the chest CT study − number (%)			0.487
Yes	2 (14)	0 (0)	
No	12 (86)	11 (100)	
Septation on the chest CT study − number (%)			0.821
Yes	7 (50)	5 (45)	
No	7 (50)	6 (55)	
Necrotic findings on the chest CT study − number (%)			0.234
Yes	6 (43)	2 (18)	
No	8 (57)	9 (82)	
Hematologic and microbiologic findings			
WBC (per μL)			0.146
Median	18	16	
IQR	17–26	8–24	
CRP (mg/L)			0.244
Median	252	142	
IQR	160–319	69–295	
Albumin (g/L)			0.012
Median	2	2.7	
IQR	1.2–2.2	2–3.2	
Positive pleural culture, number (%)			0.549
Yes	3 (19)	0 (0)	
No	13 (81)	5 (100)	
Medical and surgical management			
The initial antibiotic changed during hospitalization − number (%)			1.00
Yes	12 (75)	10 (71)	
No	4 (25)	4 (29)	
Oral antibiotics given on discharge − number (%)			0.586
Yes	15 (94)	12 (86)	
No	1 (6)	2 (4)	
Duration of IV antibiotics (days)			0.234
Median	20	13	
IQR	13–21	9–25	
Duration of oral antibiotics (days)			0.025
Median	21	14	
IQR	19–26	10–14	
Duration of hospitalization (days)			0.382
Median	13	14	
IQR	8–20	7–24	
Normal findings on the following chest X-ray after 3 months, number (%)			1.00
Yes	7 (64)	4 (67)	
No	4 (36)	2 (33)	
Lung consolidation on the following chest X-ray after 3 months, number (%)			1.00
Yes	3 (27)	2 (33)	
No	8 (73)	4 (67)	

## Discussion

The purpose of this study is to describe the demographic features, clinical presentation, management, and outcome of patients diagnosed with CCAP at AQWCH. The study group comprises children admitted to AQWCH diagnosed with CCAP between January 01, 2018, and December 31, 2020. The diagnosis of CCAP was made based on the clinical, radiological, and hematological findings. CCAP in previously healthy children was found to be associated with younger age, less than two years, a long duration of fever before admission, asymmetric chest pain, high inflammatory markers, low WBC, iron-deficiency anemia, and pretreatment with analgesic medications [10-14]. Similarly, our study showed that the median age was 2.5 years and the median duration of stay in the hospital was 16 days, with a longer duration of fever and high inflammatory markers.

In our study, the incidence of CCAP was found to be 15.3%. Of these, NP was 46.6%, PPE was 26.7%, and EMP was 26.7%. A higher incidence of complicated pneumonia compared with 3% of the British Association pediatric pneumonia audit [15] was found as a result of the delay in diagnosing and transferring complicated cases from other health facilities. A retrospective, observational study conducted at Boston Children’s Hospital over 15 years showed an increased incidence of NP [16].

In this study, all patients had been vaccinated with PCV13 or PCV7 and Hib; however, blood culture was negative in all cases and pleural culture was positive only in three (14%) cases, which may have been due to the antibiotic use before admission or technical issues (methods of blood collection and quantities of blood collected). The retrospective study for EMP conducted in Canada from eight pediatric hospitals showed that 56/88 (63.6%) patients had positive pleural cultures [17], and a study conducted in North America showed that empirical antibiotic use decreases the positive cultures from 60% to 30% [18]. In another study conducted in six children’s hospitals in the United States for patients hospitalized with CAP, a positive blood culture was obtained (2-5%) [19]. One of the detailed studies carried out before the introduction of PCV13 in the United States, supported by European studies after the introduction of PCV13, had shown that *S. pneumoniae* was the most common cause of CCAP. However, immunization with PCV13 was linked to decreased invasive pneumococcal disease and increased incidence of other organisms, especially *S. pyogenes* [20-23].

Imaging studies are important tools in CCAP. The chest X-ray helps in diagnosing diseases in patients, but there are chances of missed diagnosis in some patients, or the diagnosis may not differentiate between abscess formation and other thoracic malformations or consolidation [3]. Chest ultrasound is more sensitive than chest X-ray for detecting small effusions, differentiating and diagnosing septation, consolidation, lung abscess, and EMP [24,25]. CT does not provide a better diagnosis from chest ultrasound in CCAP in terms of planning its management and predicting outcomes. The CT should be used for complex cases to guide intervention and unclear diagnosis, or if there is no improvement in disease management [26], which is observed in this study.

Interestingly, in this study, PICU admission was high at 70% and low at 2% in the British Association pediatric pneumonia audit [15], and most patients were admitted preoperatively (67%). This was followed as there are no high-dependency care units in our facility. On analyzing the data for different types of CCAP, it was found that the duration of hospital stay for NP was shorter compared with other studies that ranged between 13.5 days and 27 days. Most patients underwent early surgical intervention (VATS) [27,28].

The surgical treatment for EMP is still disputable. VATS allows pleural debridement and drains pus from the pleural cavity under direct vision. VATS needs a specifically trained surgeon, which is unavailable in most centers. In both retrospective and prospective uncontrolled studies, VATS had a better result than chest tube drainage alone in resolving EMP [29]. In our study, four patients required a chest tube, and two underwent VATS later during the hospitalization. The complications reported with VATS are bronchopleural fistula, lung injury, and death [30]. In this study, most complications post-VATS were surgical emphysema (65%), pneumothorax (24%), and bronchopulmonary fistula (8%). Unfortunately, one (16.7%) patient of the total CCAP patients with NP died despite the required management and care taken in the PICU.

Strengths, limitations, and generalizability

This is a retrospective chart review study; many positive and significant statistical analysis results were found, which is the main strength of the study. Due to the small sample size, some variables could not be analyzed in this study. The data abstractors failed to develop the study proposal and protocol to minimize biases. In other words, they failed to understand the study objectives and research questions. The study was performed in a tertiary pediatric hospital where cases were referred from other hospitals, mostly with complications. Hence, this population did not match the general population. The incidence of CCAP for the whole UAE cannot be determined from this data.

One of the main limitations of this study was the lack of references and articles related to this subject, especially in the UAE, for comparing our study with the published research papers. Another major limitation was the non-availability of preadmission units for patients, which directly affected the progress of the treatment of CCAP.

The Responsible Conduct of Research (RCR) is considered protected health information that will not be revealed to anybody. However, the data may be used for future prospective studies, official monitoring, supervision, or if the law requires it.

## Conclusions

CCAP remains a major cause of hospitalization in children. It is important to suspect developed CCAP in all CAP patients who do not respond to treatment after 48-72 hours. The imaging study is important in both diagnosis and intervention. The cornerstone of treatment is antibiotic therapy. The surgical option should be kept for the selected patient, and the decision should be taken on a case-by-case basis. Most patients are cured without any long-term complications; however, there is mortality in severe cases.
